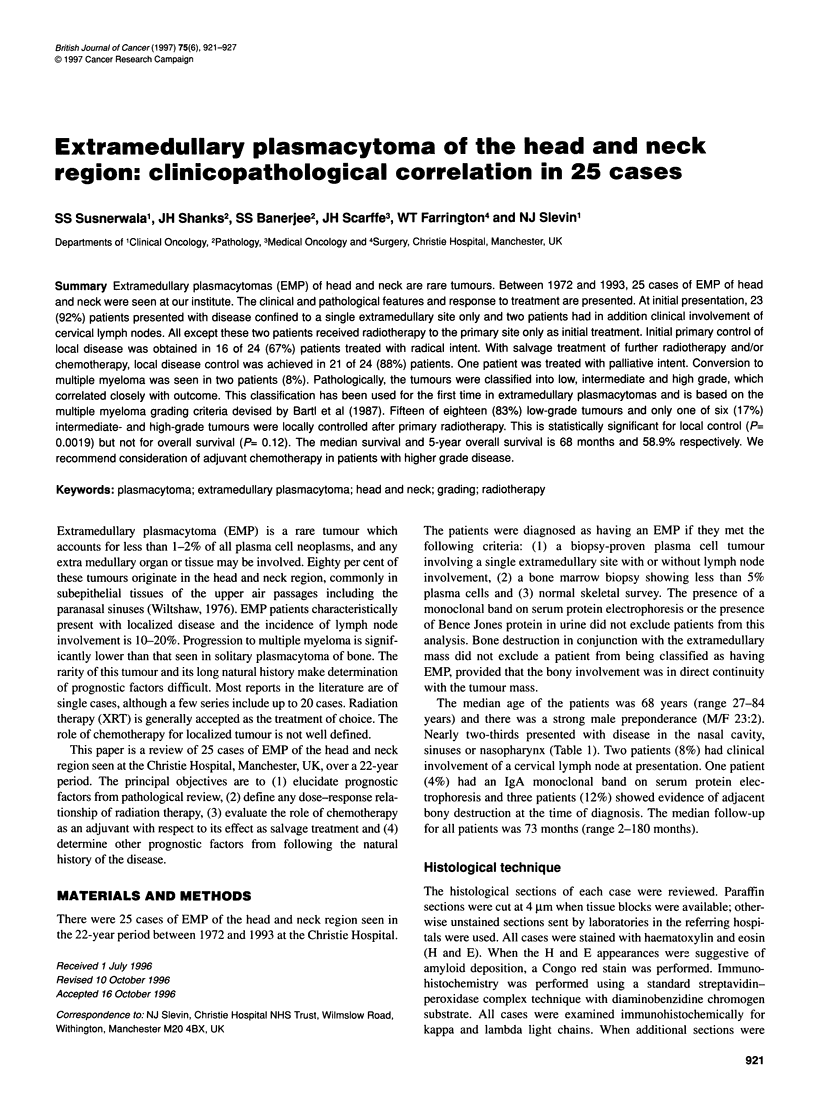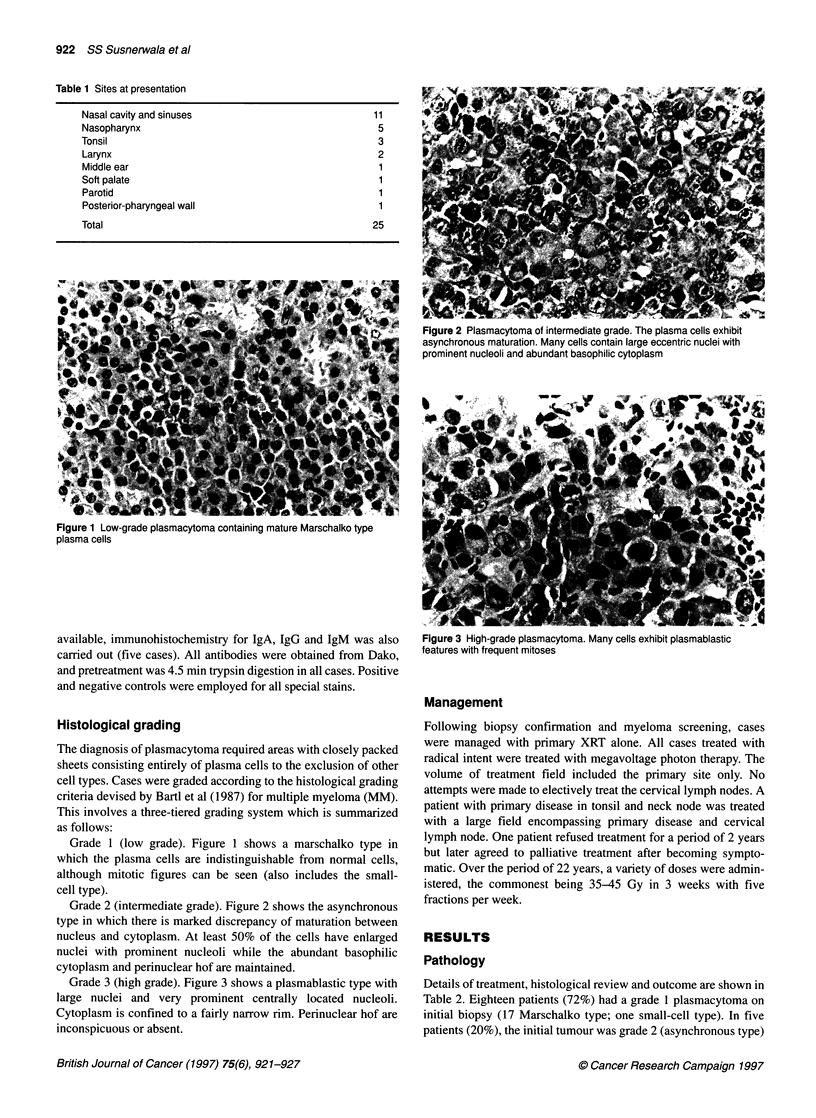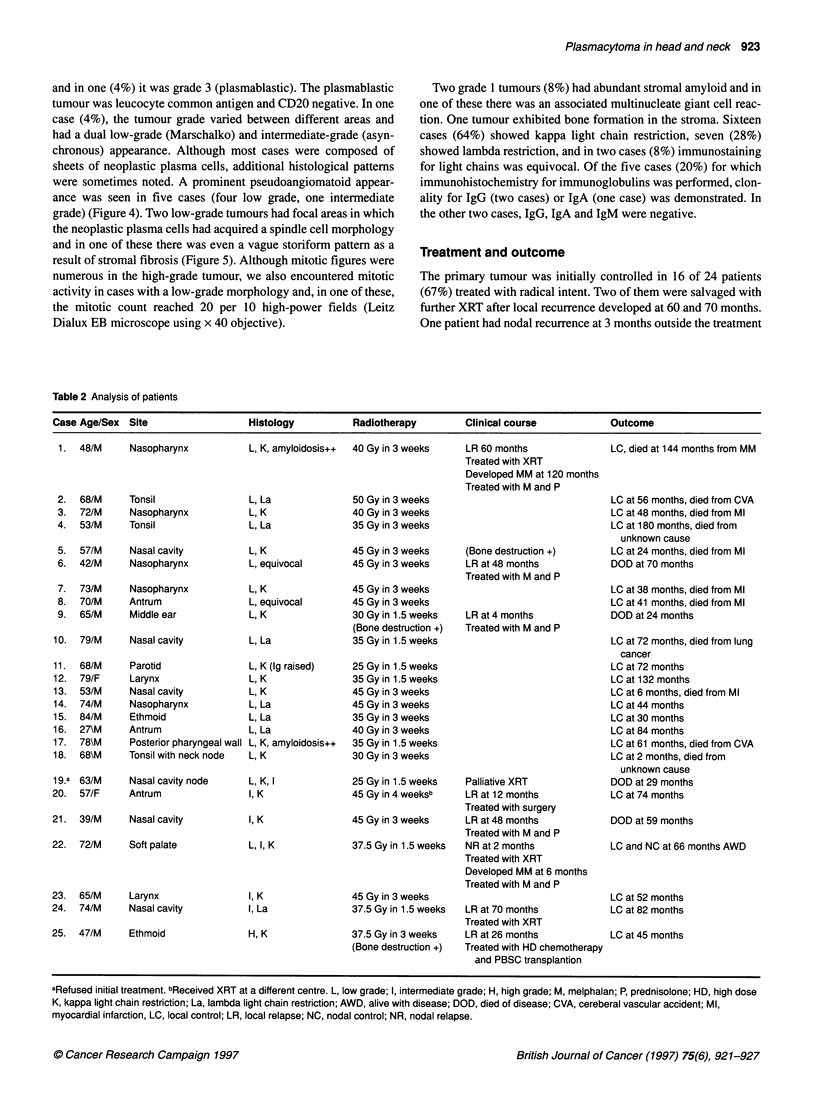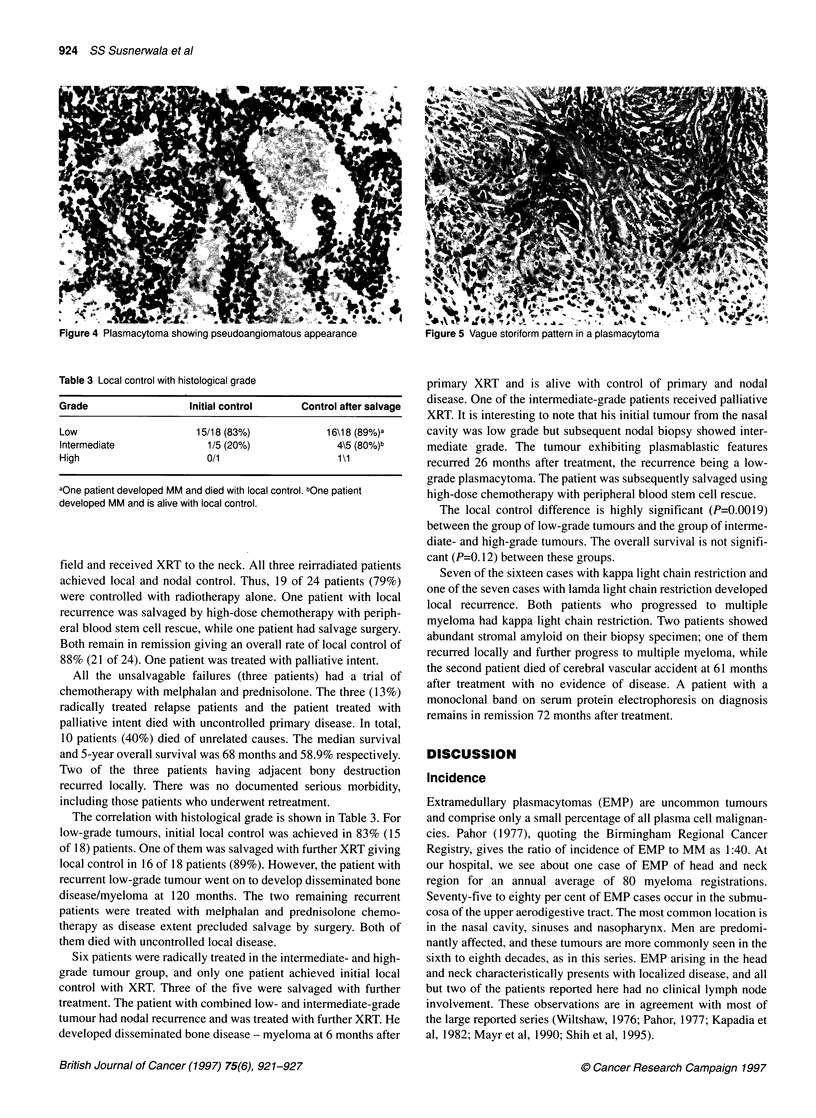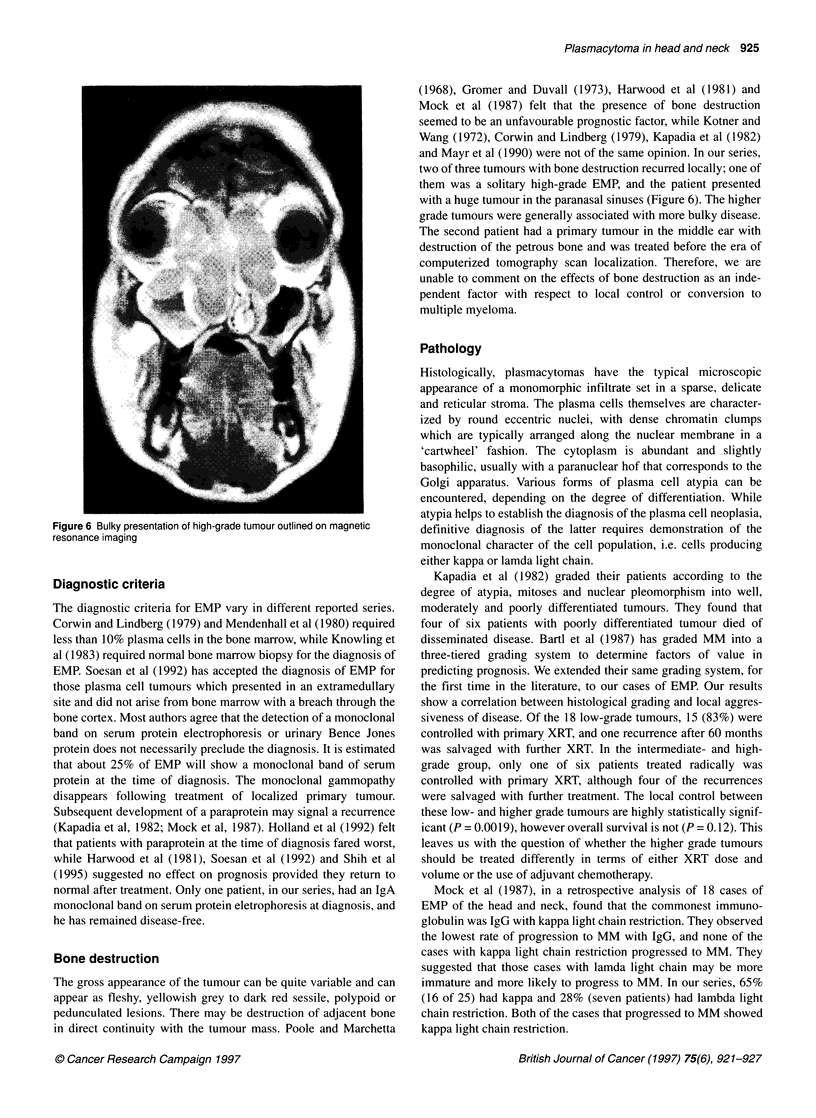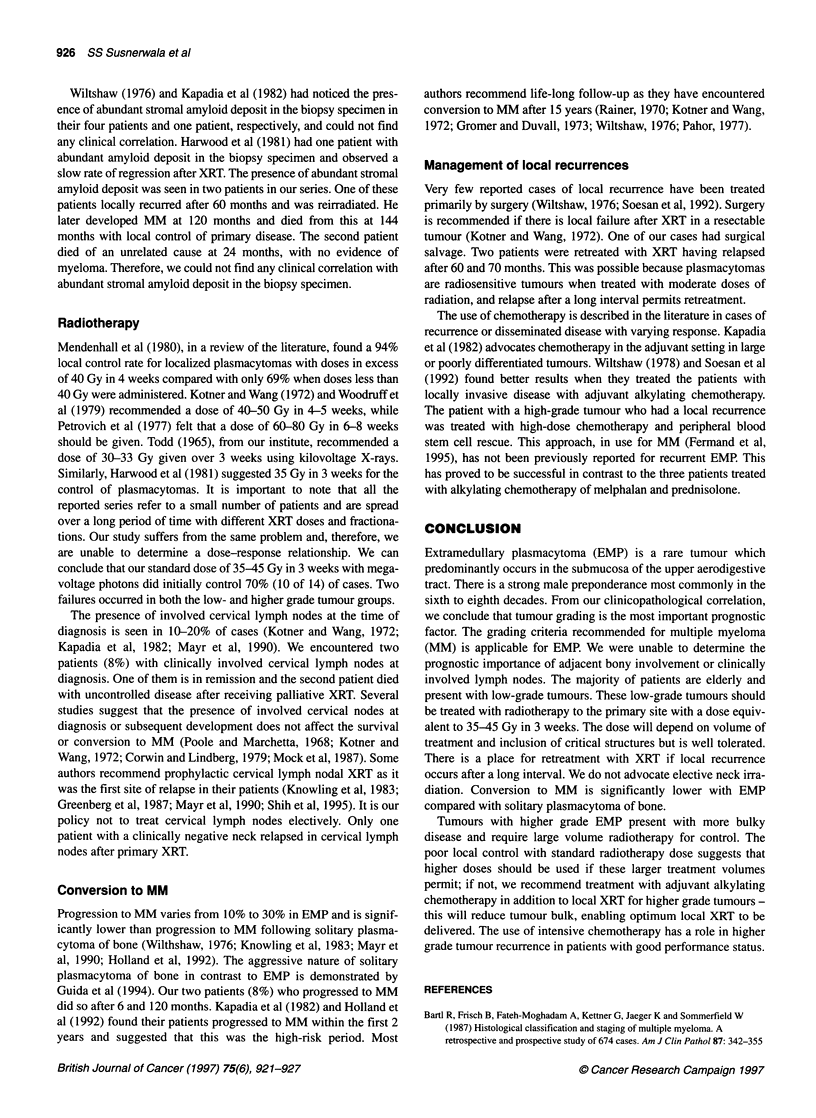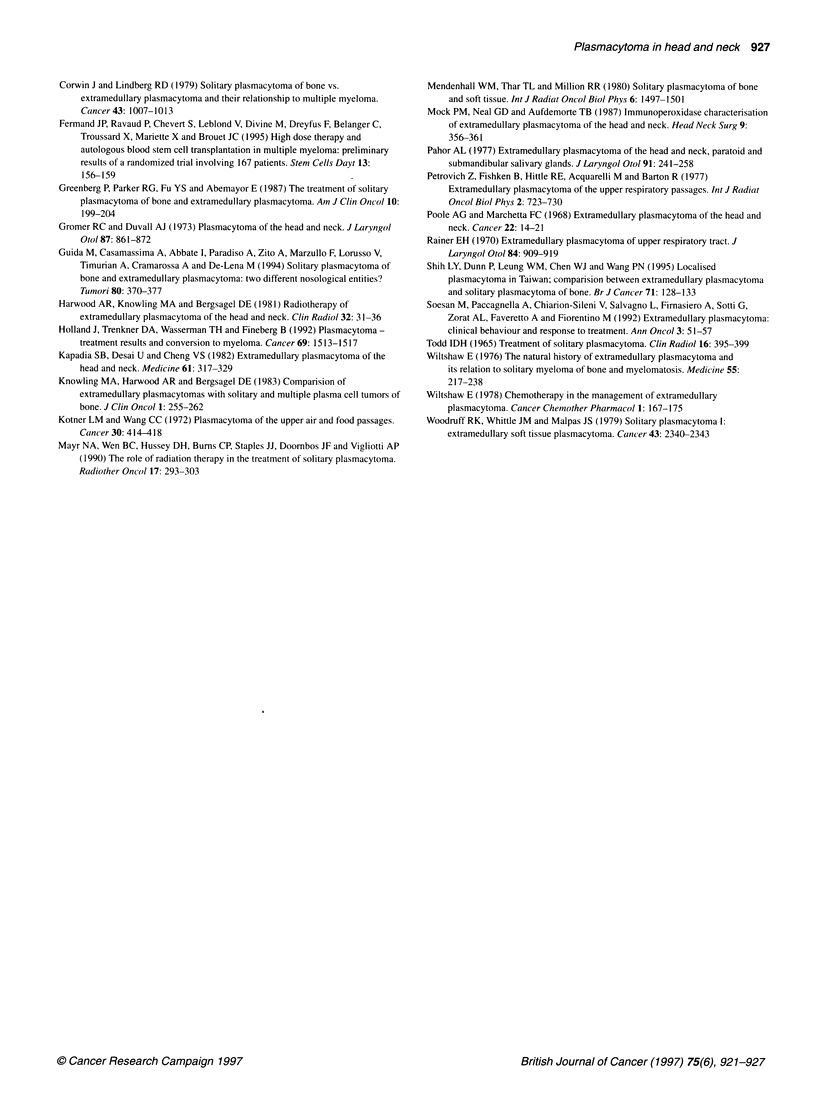# Extramedullary plasmacytoma of the head and neck region: clinicopathological correlation in 25 cases.

**DOI:** 10.1038/bjc.1997.162

**Published:** 1997

**Authors:** S. S. Susnerwala, J. H. Shanks, S. S. Banerjee, J. H. Scarffe, W. T. Farrington, N. J. Slevin

**Affiliations:** Department of Clinical Oncology, Christie Hospital, Manchester, UK.

## Abstract

**Images:**


British Journal of Cancer (1997) 75(6), 921–927
© 1997 Cancer Research Campaign

### Extramedullary plasmacytoma of the head and neck region: clinicopathological correlation in 25 cases

**SS Susnerwala[1], JH Shanks[2], SS Banerjee[2], JH Scarffe[3], WT Farrington[4] and NJ Slevin[1]**

Departments of [1]Clinical Oncology, [2]Pathology, [3]Medical Oncology and [4]Surgery, Christie Hospital, Manchester, UK

**Summary** Extramedullary plasmacytomas (EMP) of head and neck are rare tumours. Between 1972 and 1993, 25 cases of EMP of head and neck were seen at our institute. The clinical and pathological features and response to treatment are presented. At initial presentation, 23 (92%) patients presented with disease confined to a single extramedullary site only and two patients had in addition clinical involvement of cervical lymph nodes. All except these two patients received radiotherapy to the primary site only as initial treatment. Initial primary control of local disease was obtained in 16 of 24 (67%) patients treated with radical intent. With salvage treatment of further radiotherapy and/or chemotherapy, local disease control was achieved in 21 of 24 (88%) patients. One patient was treated with palliative intent. Conversion to multiple myeloma was seen in two patients (8%). Pathologically, the tumours were classified into low, intermediate and high grade, which correlated closely with outcome. This classification has been used for the first time in extramedullary plasmacytomas and is based on the multiple myeloma grading criteria devised by Bartl et al (1987). Fifteen of eighteen (83%) low-grade tumours and only one of six (17%) intermediate- and high-grade tumours were locally controlled after primary radiotherapy. This is statistically significant for local control (*P*= 0.0019) but not for overall survival (*P*= 0.12). The median survival and 5-year overall survival is 68 months and 58.9% respectively. We recommend consideration of adjuvant chemotherapy in patients with higher grade disease.

**Keywords:** plasmacytoma; extramedullary plasmacytoma; head and neck; grading; radiotherapy

Extramedullary plasmacytoma (EMP) is a rare tumour which accounts for less than 1–2% of all plasma cell neoplasms, and any extra medullary organ or tissue may be involved. Eighty per cent of these tumours originate in the head and neck region, commonly in subepithelial tissues of the upper air passages including the paranasal sinuses (Wiltshaw, 1976). EMP patients characteristically present with localized disease and the incidence of lymph node involvement is 10–20%. Progression to multiple myeloma is significantly lower than that seen in solitary plasmacytoma of bone. The rarity of this tumour and its long natural history make determination of prognostic factors difficult. Most reports in the literature are of single cases, although a few series include up to 20 cases. Radiation therapy (XRT) is generally accepted as the treatment of choice. The role of chemotherapy for localized tumour is not well defined.

This paper is a review of 25 cases of EMP of the head and neck region seen at the Christie Hospital, Manchester, UK, over a 22-year period. The principal objectives are to (1) elucidate prognostic factors from pathological review, (2) define any dose–response relationship of radiation therapy, (3) evaluate the role of chemotherapy as an adjuvant with respect to its effect as salvage treatment and (4) determine other prognostic factors from following the natural history of the disease.

### MATERIALS AND METHODS

There were 25 cases of EMP of the head and neck region seen in the 22-year period between 1972 and 1993 at the Christie Hospital.

*Received 1 July 1996*
*Revised 10 October 1996*
*Accepted 16 October 1996*

*Correspondence to:* NJ Slevin, Christie Hospital NHS Trust, Wilmslow Road, Withington, Manchester M20 4BX, UK

The patients were diagnosed as having an EMP if they met the following criteria: (1) a biopsy-proven plasma cell tumour involving a single extramedullary site with or without lymph node involvement, (2) a bone marrow biopsy showing less than 5% plasma cells and (3) normal skeletal survey. The presence of a monoclonal band on serum protein electrophoresis or the presence of Bence Jones protein in urine did not exclude patients from this analysis. Bone destruction in conjunction with the extramedullary mass did not exclude a patient from being classified as having EMP, provided that the bony involvement was in direct continuity with the tumour mass.

The median age of the patients was 68 years (range 27–84 years) and there was a strong male preponderance (M/F 23:2). Nearly two-thirds presented with disease in the nasal cavity, sinuses or nasopharynx (Table 1). Two patients (8%) had clinical involvement of a cervical lymph node at presentation. One patient (4%) had an IgA monoclonal band on serum protein electrophoresis and three patients (12%) showed evidence of adjacent bony destruction at the time of diagnosis. The median follow-up for all patients was 73 months (range 2–180 months).

### Histological technique

The histological sections of each case were reviewed. Paraffin sections were cut at 4 μm when tissue blocks were available; otherwise unstained sections sent by laboratories in the referring hospitals were used. All cases were stained with haematoxylin and eosin (H and E). When the H and E appearances were suggestive of amyloid deposition, a Congo red stain was performed. Immunohistochemistry was performed using a standard streptavidin–peroxidase complex technique with diaminobenzidine chromogen substrate. All cases were examined immunohistochemically for kappa and lambda light chains. When additional sections were

Table 1 Sites at presentation

| | |
|---|---|
| Nasal cavity and sinuses | 11 |
| Nasopharynx | 5 |
| Tonsil | 3 |
| Larynx | 2 |
| Middle ear | 1 |
| Soft palate | 1 |
| Parotid | 1 |
| Posterior-pharyngeal wall | 1 |
| Total | 25 |

**Figure 1** Low-grade plasmacytoma containing mature Marschalko type plasma cells

**Figure 2** Plasmacytoma of intermediate grade. The plasma cells exhibit asynchronous maturation. Many cells contain large eccentric nuclei with prominent nucleoli and abundant basophilic cytoplasm

**Figure 3** High-grade plasmacytoma. Many cells exhibit plasmablastic features with frequent mitoses available, immunohistochemistry for IgA, IgG and IgM was also carried out (five cases). All antibodies were obtained from Dako, and pretreatment was 4.5 min trypsin digestion in all cases. Positive and negative controls were employed for all special stains.

### Histological grading

The diagnosis of plasmacytoma required areas with closely packed sheets consisting entirely of plasma cells to the exclusion of other cell types. Cases were graded according to the histological grading criteria devised by Bartl et al (1987) for multiple myeloma (MM). This involves a three-tiered grading system which is summarized as follows:

Grade 1 (low grade). Figure 1 shows a marschalko type in which the plasma cells are indistinguishable from normal cells, although mitotic figures can be seen (also includes the small-cell type).

Grade 2 (intermediate grade). Figure 2 shows the asynchronous type in which there is marked discrepancy of maturation between nucleus and cytoplasm. At least 50% of the cells have enlarged nuclei with prominent nucleoli while the abundant basophilic cytoplasm and perinuclear hof are maintained.

Grade 3 (high grade). Figure 3 shows a plasmablastic type with large nuclei and very prominent centrally located nucleoli. Cytoplasm is confined to a fairly narrow rim. Perinuclear hof are inconspicuous or absent.

### Management

Following biopsy confirmation and myeloma screening, cases were managed with primary XRT alone. All cases treated with radical intent were treated with megavoltage photon therapy. The volume of treatment field included the primary site only. No attempts were made to electively treat the cervical lymph nodes. A patient with primary disease in tonsil and neck node was treated with a large field encompassing primary disease and cervical lymph node. One patient refused treatment for a period of 2 years but later agreed to palliative treatment after becoming symptomatic. Over the period of 22 years, a variety of doses were administered, the commonest being 35–45 Gy in 3 weeks with five fractions per week.

### RESULTS

### Pathology

Details of treatment, histological review and outcome are shown in Table 2. Eighteen patients (72%) had a grade 1 plasmacytoma on initial biopsy (17 Marschalko type; one small-cell type). In five patients (20%), the initial tumour was grade 2 (asynchronous type)

© Cancer Research Campaign 1997

and in one (4%) it was grade 3 (plasmablastic). The plasmablastic tumour was leucocyte common antigen and CD20 negative. In one case (4%), the tumour grade varied between different areas and had a dual low-grade (Marschalko) and intermediate-grade (asynchronous) appearance. Although most cases were composed of sheets of neoplastic plasma cells, additional histological patterns were sometimes noted. A prominent pseudoangiomatoid appearance was seen in five cases (four low grade, one intermediate grade) (Figure 4). Two low-grade tumours had focal areas in which the neoplastic plasma cells had acquired a spindle cell morphology and in one of these there was even a vague storiform pattern as a result of stromal fibrosis (Figure 5). Although mitotic figures were numerous in the high-grade tumour, we also encountered mitotic activity in cases with a low-grade morphology and, in one of these, the mitotic count reached 20 per 10 high-power fields (Leitz Dialux EB microscope using × 40 objective).

Two grade 1 tumours (8%) had abundant stromal amyloid and in one of these there was an associated multinucleate giant cell reaction. One tumour exhibited bone formation in the stroma. Sixteen cases (64%) showed kappa light chain restriction, seven (28%) showed lambda restriction, and in two cases (8%) immunostaining for light chains was equivocal. Of the five cases (20%) for which immunohistochemistry for immunoglobulins was performed, clonality for IgG (two cases) or IgA (one case) was demonstrated. In the other two cases, IgG, IgA and IgM were negative.

### Treatment and outcome

The primary tumour was initially controlled in 16 of 24 patients (67%) treated with radical intent. Two of them were salvaged with further XRT after local recurrence developed at 60 and 70 months. One patient had nodal recurrence at 3 months outside the treatment

**Table 2** Analysis of patients

| Case | Age/Sex | Site | Histology | Radiotherapy | Clinical course | Outcome |
|---|---|---|---|---|---|---|
| 1. | 48/M | Nasopharynx | L, K, amyloidosis++ | 40 Gy in 3 weeks | LR 60 months<br>Treated with XRT<br>Developed MM at 120 months<br>Treated with M and P | LC, died at 144 months from MM |
| 2. | 68/M | Tonsil | L, La | 50 Gy in 3 weeks | | LC at 56 months, died from CVA |
| 3. | 72/M | Nasopharynx | L, K | 40 Gy in 3 weeks | | LC at 48 months, died from MI |
| 4. | 53/M | Tonsil | L, La | 35 Gy in 3 weeks | | LC at 180 months, died from unknown cause |
| 5. | 57/M | Nasal cavity | L, K | 45 Gy in 3 weeks | (Bone destruction +) | LC at 24 months, died from MI |
| 6. | 42/M | Nasopharynx | L, equivocal | 45 Gy in 3 weeks | LR at 48 months<br>Treated with M and P | DOD at 70 months |
| 7. | 73/M | Nasopharynx | L, K | 45 Gy in 3 weeks | | LC at 38 months, died from MI |
| 8. | 70/M | Antrum | L, equivocal | 45 Gy in 3 weeks | | LC at 41 months, died from MI |
| 9. | 65/M | Middle ear | L, K | 30 Gy in 1.5 weeks<br>(Bone destruction +) | LR at 4 months<br>Treated with M and P | DOD at 24 months |
| 10. | 79/M | Nasal cavity | L, La | 35 Gy in 1.5 weeks | | LC at 72 months, died from lung cancer |
| 11. | 68/M | Parotid | L, K (Ig raised) | 25 Gy in 1.5 weeks | | LC at 72 months |
| 12. | 79/F | Larynx | L, K | 35 Gy in 1.5 weeks | | LC at 132 months |
| 13. | 53/M | Nasal cavity | L, K | 45 Gy in 3 weeks | | LC at 6 months, died from MI |
| 14. | 74/M | Nasopharynx | L, La | 45 Gy in 3 weeks | | LC at 44 months |
| 15. | 84/M | Ethmoid | L, La | 35 Gy in 3 weeks | | LC at 30 months |
| 16. | 27\M | Antrum | L, La | 40 Gy in 3 weeks | | LC at 84 months |
| 17. | 78\M | Posterior pharyngeal wall | L, K, amyloidosis++ | 35 Gy in 1.5 weeks | | LC at 61 months, died from CVA |
| 18. | 68\M | Tonsil with neck node | L, K | 30 Gy in 3 weeks | | LC at 2 months, died from unknown cause |
| 19.[a] | 63/M | Nasal cavity node | L, K, I | 25 Gy in 1.5 weeks | Palliative XRT | DOD at 29 months |
| 20. | 57/F | Antrum | I, K | 45 Gy in 4 weeks[b] | LR at 12 months<br>Treated with surgery | LC at 74 months |
| 21. | 39/M | Nasal cavity | I, K | 45 Gy in 3 weeks | LR at 48 months<br>Treated with M and P | DOD at 59 months |
| 22. | 72/M | Soft palate | L, I, K | 37.5 Gy in 1.5 weeks | NR at 2 months<br>Treated with XRT<br>Developed MM at 6 months<br>Treated with M and P | LC and NC at 66 months AWD |
| 23. | 65/M | Larynx | I, K | 45 Gy in 3 weeks | | LC at 52 months |
| 24. | 74/M | Nasal cavity | I, La | 37.5 Gy in 1.5 weeks | LR at 70 months<br>Treated with XRT | LC at 82 months |
| 25. | 47/M | Ethmoid | H, K | 37.5 Gy in 3 weeks<br>(Bone destruction +) | LR at 26 months<br>Treated with HD chemotherapy and PBSC transplantion | LC at 45 months |

[a]Refused initial treatment. [b]Received XRT at a different centre. L, low grade; I, intermediate grade; H, high grade; M, melphalan; P, prednisolone; HD, high dose K, kappa light chain restriction; La, lambda light chain restriction; AWD, alive with disease; DOD, died of disease; CVA, cereberal vascular accident; MI, myocardial infarction, LC, local control; LR, local relapse; NC, nodal control; NR, nodal relapse.

© *Cancer Research Campaign 1997*

**Figure 4** Plasmacytoma showing pseudoangiomatous appearance

**Figure 5** Vague storiform pattern in a plasmacytoma

**Table 3** Local control with histological grade

| Grade | Initial control | Control after salvage |
|---|---|---|
| Low | 15/18 (83%) | 16\18 (89%)[a] |
| Intermediate | 1/5 (20%) | 4\5 (80%)[b] |
| High | 0/1 | 1\1 |

[a]One patient developed MM and died with local control. [b]One patient developed MM and is alive with local control.

field and received XRT to the neck. All three reirradiated patients achieved local and nodal control. Thus, 19 of 24 patients (79%) were controlled with radiotherapy alone. One patient with local recurrence was salvaged by high-dose chemotherapy with peripheral blood stem cell rescue, while one patient had salvage surgery. Both remain in remission giving an overall rate of local control of 88% (21 of 24). One patient was treated with palliative intent.

All the unsalvagable failures (three patients) had a trial of chemotherapy with melphalan and prednisolone. The three (13%) radically treated relapse patients and the patient treated with palliative intent died with uncontrolled primary disease. In total, 10 patients (40%) died of unrelated causes. The median survival and 5-year overall survival was 68 months and 58.9% respectively. Two of the three patients having adjacent bony destruction recurred locally. There was no documented serious morbidity, including those patients who underwent retreatment.

The correlation with histological grade is shown in Table 3. For low-grade tumours, initial local control was achieved in 83% (15 of 18) patients. One of them was salvaged with further XRT giving local control in 16 of 18 patients (89%). However, the patient with recurrent low-grade tumour went on to develop disseminated bone disease/myeloma at 120 months. The two remaining recurrent patients were treated with melphalan and prednisolone chemotherapy as disease extent precluded salvage by surgery. Both of them died with uncontrolled local disease.

Six patients were radically treated in the intermediate- and high-grade tumour group, and only one patient achieved initial local control with XRT. Three of the five were salvaged with further treatment. The patient with combined low- and intermediate-grade tumour had nodal recurrence and was treated with further XRT. He developed disseminated bone disease – myeloma at 6 months after primary XRT and is alive with control of primary and nodal disease. One of the intermediate-grade patients received palliative XRT. It is interesting to note that his initial tumour from the nasal cavity was low grade but subsequent nodal biopsy showed intermediate grade. The tumour exhibiting plasmablastic features recurred 26 months after treatment, the recurrence being a low-grade plasmacytoma. The patient was subsequently salvaged using high-dose chemotherapy with peripheral blood stem cell rescue.

The local control difference is highly significant ($P$=0.0019) between the group of low-grade tumours and the group of intermediate- and high-grade tumours. The overall survival is not significant ($P$=0.12) between these groups.

Seven of the sixteen cases with kappa light chain restriction and one of the seven cases with lamda light chain restriction developed local recurrence. Both patients who progressed to multiple myeloma had kappa light chain restriction. Two patients showed abundant stromal amyloid on their biopsy specimen; one of them recurred locally and further progress to multiple myeloma, while the second patient died of cerebral vascular accident at 61 months after treatment with no evidence of disease. A patient with a monoclonal band on serum protein electrophoresis on diagnosis remains in remission 72 months after treatment.

### DISCUSSION

### Incidence

Extramedullary plasmacytomas (EMP) are uncommon tumours and comprise only a small percentage of all plasma cell malignancies. Pahor (1977), quoting the Birmingham Regional Cancer Registry, gives the ratio of incidence of EMP to MM as 1:40. At our hospital, we see about one case of EMP of head and neck region for an annual average of 80 myeloma registrations. Seventy-five to eighty per cent of EMP cases occur in the submucosa of the upper aerodigestive tract. The most common location is in the nasal cavity, sinuses and nasopharynx. Men are predominantly affected, and these tumours are more commonly seen in the sixth to eighth decades, as in this series. EMP arising in the head and neck characteristically presents with localized disease, and all but two of the patients reported here had no clinical lymph node involvement. These observations are in agreement with most of the large reported series (Wiltshaw, 1976; Pahor, 1977; Kapadia et al, 1982; Mayr et al, 1990; Shih et al, 1995).

© Cancer Research Campaign 1997

**Figure 6** Bulky presentation of high-grade tumour outlined on magnetic resonance imaging

### Diagnostic criteria

The diagnostic criteria for EMP vary in different reported series. Corwin and Lindberg (1979) and Mendenhall et al (1980) required less than 10% plasma cells in the bone marrow, while Knowling et al (1983) required normal bone marrow biopsy for the diagnosis of EMP. Soesan et al (1992) has accepted the diagnosis of EMP for those plasma cell tumours which presented in an extramedullary site and did not arise from bone marrow with a breach through the bone cortex. Most authors agree that the detection of a monoclonal band on serum protein electrophoresis or urinary Bence Jones protein does not necessarily preclude the diagnosis. It is estimated that about 25% of EMP will show a monoclonal band of serum protein at the time of diagnosis. The monoclonal gammopathy disappears following treatment of localized primary tumour. Subsequent development of a paraprotein may signal a recurrence (Kapadia et al, 1982; Mock et al, 1987). Holland et al (1992) felt that patients with paraprotein at the time of diagnosis fared worst, while Harwood et al (1981), Soesan et al (1992) and Shih et al (1995) suggested no effect on prognosis provided they return to normal after treatment. Only one patient, in our series, had an IgA monoclonal band on serum protein eletrophoresis at diagnosis, and he has remained disease-free.

### Bone destruction

The gross appearance of the tumour can be quite variable and can appear as fleshy, yellowish grey to dark red sessile, polypoid or pedunculated lesions. There may be destruction of adjacent bone in direct continuity with the tumour mass. Poole and Marchetta (1968), Gromer and Duvall (1973), Harwood et al (1981) and Mock et al (1987) felt that the presence of bone destruction seemed to be an unfavourable prognostic factor, while Kotner and Wang (1972), Corwin and Lindberg (1979), Kapadia et al (1982) and Mayr et al (1990) were not of the same opinion. In our series, two of three tumours with bone destruction recurred locally; one of them was a solitary high-grade EMP, and the patient presented with a huge tumour in the paranasal sinuses (Figure 6). The higher grade tumours were generally associated with more bulky disease. The second patient had a primary tumour in the middle ear with destruction of the petrous bone and was treated before the era of computerized tomography scan localization. Therefore, we are unable to comment on the effects of bone destruction as an independent factor with respect to local control or conversion to multiple myeloma.

### Pathology

Histologically, plasmacytomas have the typical microscopic appearance of a monomorphic infiltrate set in a sparse, delicate and reticular stroma. The plasma cells themselves are characterized by round eccentric nuclei, with dense chromatin clumps which are typically arranged along the nuclear membrane in a 'cartwheel' fashion. The cytoplasm is abundant and slightly basophilic, usually with a paranuclear hof that corresponds to the Golgi apparatus. Various forms of plasma cell atypia can be encountered, depending on the degree of differentiation. While atypia helps to establish the diagnosis of the plasma cell neoplasia, definitive diagnosis of the latter requires demonstration of the monoclonal character of the cell population, i.e. cells producing either kappa or lamda light chain.

Kapadia et al (1982) graded their patients according to the degree of atypia, mitoses and nuclear pleomorphism into well, moderately and poorly differentiated tumours. They found that four of six patients with poorly differentiated tumour died of disseminated disease. Bartl et al (1987) has graded MM into a three-tiered grading system to determine factors of value in predicting prognosis. We extended their same grading system, for the first time in the literature, to our cases of EMP. Our results show a correlation between histological grading and local aggressiveness of disease. Of the 18 low-grade tumours, 15 (83%) were controlled with primary XRT, and one recurrence after 60 months was salvaged with further XRT. In the intermediate- and high-grade group, only one of six patients treated radically was controlled with primary XRT, although four of the recurrences were salvaged with further treatment. The local control between these low- and higher grade tumours are highly statistically significant ($P = 0.0019$), however overall survival is not ($P = 0.12$). This leaves us with the question of whether the higher grade tumours should be treated differently in terms of either XRT dose and volume or the use of adjuvant chemotherapy.

Mock et al (1987), in a retrospective analysis of 18 cases of EMP of the head and neck, found that the commonest immunoglobulin was IgG with kappa light chain restriction. They observed the lowest rate of progression to MM with IgG, and none of the cases with kappa light chain restriction progressed to MM. They suggested that those cases with lamda light chain may be more immature and more likely to progress to MM. In our series, 65% (16 of 25) had kappa and 28% (seven patients) had lambda light chain restriction. Both of the cases that progressed to MM showed kappa light chain restriction.

Wiltshaw (1976) and Kapadia et al (1982) had noticed the presence of abundant stromal amyloid deposit in the biopsy specimen in their four patients and one patient, respectively, and could not find any clinical correlation. Harwood et al (1981) had one patient with abundant amyloid deposit in the biopsy specimen and observed a slow rate of regression after XRT. The presence of abundant stromal amyloid deposit was seen in two patients in our series. One of these patients locally recurred after 60 months and was reirradiated. He later developed MM at 120 months and died from this at 144 months with local control of primary disease. The second patient died of an unrelated cause at 24 months, with no evidence of myeloma. Therefore, we could not find any clinical correlation with abundant stromal amyloid deposit in the biopsy specimen.

### Radiotherapy

Mendenhall et al (1980), in a review of the literature, found a 94% local control rate for localized plasmacytomas with doses in excess of 40 Gy in 4 weeks compared with only 69% when doses less than 40 Gy were administered. Kotner and Wang (1972) and Woodruff et al (1979) recommended a dose of 40–50 Gy in 4–5 weeks, while Petrovich et al (1977) felt that a dose of 60–80 Gy in 6–8 weeks should be given. Todd (1965), from our institute, recommended a dose of 30–33 Gy given over 3 weeks using kilovoltage X-rays. Similarly, Harwood et al (1981) suggested 35 Gy in 3 weeks for the control of plasmacytomas. It is important to note that all the reported series refer to a small number of patients and are spread over a long period of time with different XRT doses and fractionations. Our study suffers from the same problem and, therefore, we are unable to determine a dose–response relationship. We can conclude that our standard dose of 35–45 Gy in 3 weeks with megavoltage photons did initially control 70% (10 of 14) of cases. Two failures occurred in both the low- and higher grade tumour groups.

The presence of involved cervical lymph nodes at the time of diagnosis is seen in 10–20% of cases (Kotner and Wang, 1972; Kapadia et al, 1982; Mayr et al, 1990). We encountered two patients (8%) with clinically involved cervical lymph nodes at diagnosis. One of them is in remission and the second patient died with uncontrolled disease after receiving palliative XRT. Several studies suggest that the presence of involved cervical nodes at diagnosis or subsequent development does not affect the survival or conversion to MM (Poole and Marchetta, 1968; Kotner and Wang, 1972; Corwin and Lindberg, 1979; Mock et al, 1987). Some authors recommend prophylactic cervical lymph nodal XRT as it was the first site of relapse in their patients (Knowling et al, 1983; Greenberg et al, 1987; Mayr et al, 1990; Shih et al, 1995). It is our policy not to treat cervical lymph nodes electively. Only one patient with a clinically negative neck relapsed in cervical lymph nodes after primary XRT.

### Conversion to MM

Progression to MM varies from 10% to 30% in EMP and is significantly lower than progression to MM following solitary plasmacytoma of bone (Wilthshaw, 1976; Knowling et al, 1983; Mayr et al, 1990; Holland et al, 1992). The aggressive nature of solitary plasmacytoma of bone in contrast to EMP is demonstrated by Guida et al (1994). Our two patients (8%) who progressed to MM did so after 6 and 120 months. Kapadia et al (1982) and Holland et al (1992) found their patients progressed to MM within the first 2 years and suggested that this was the high-risk period. Most authors recommend life-long follow-up as they have encountered conversion to MM after 15 years (Rainer, 1970; Kotner and Wang, 1972; Gromer and Duvall, 1973; Wiltshaw, 1976; Pahor, 1977).

### Management of local recurrences

Very few reported cases of local recurrence have been treated primarily by surgery (Wiltshaw, 1976; Soesan et al, 1992). Surgery is recommended if there is local failure after XRT in a resectable tumour (Kotner and Wang, 1972). One of our cases had surgical salvage. Two patients were retreated with XRT having relapsed after 60 and 70 months. This was possible because plasmacytomas are radiosensitive tumours when treated with moderate doses of radiation, and relapse after a long interval permits retreatment.

The use of chemotherapy is described in the literature in cases of recurrence or disseminated disease with varying response. Kapadia et al (1982) advocates chemotherapy in the adjuvant setting in large or poorly differentiated tumours. Wiltshaw (1978) and Soesan et al (1992) found better results when they treated the patients with locally invasive disease with adjuvant alkylating chemotherapy. The patient with a high-grade tumour who had a local recurrence was treated with high-dose chemotherapy and peripheral blood stem cell rescue. This approach, in use for MM (Fermand et al, 1995), has not been previously reported for recurrent EMP. This has proved to be successful in contrast to the three patients treated with alkylating chemotherapy of melphalan and prednisolone.

### CONCLUSION

Extramedullary plasmacytoma (EMP) is a rare tumour which predominantly occurs in the submucosa of the upper aerodigestive tract. There is a strong male preponderance most commonly in the sixth to eighth decades. From our clinicopathological correlation, we conclude that tumour grading is the most important prognostic factor. The grading criteria recommended for multiple myeloma (MM) is applicable for EMP. We were unable to determine the prognostic importance of adjacent bony involvement or clinically involved lymph nodes. The majority of patients are elderly and present with low-grade tumours. These low-grade tumours should be treated with radiotherapy to the primary site with a dose equivalent to 35–45 Gy in 3 weeks. The dose will depend on volume of treatment and inclusion of critical structures but is well tolerated. There is a place for retreatment with XRT if local recurrence occurs after a long interval. We do not advocate elective neck irradiation. Conversion to MM is significantly lower with EMP compared with solitary plasmacytoma of bone.

Tumours with higher grade EMP present with more bulky disease and require large volume radiotherapy for control. The poor local control with standard radiotherapy dose suggests that higher doses should be used if these larger treatment volumes permit; if not, we recommend treatment with adjuvant alkylating chemotherapy in addition to local XRT for higher grade tumours – this will reduce tumour bulk, enabling optimum local XRT to be delivered. The use of intensive chemotherapy has a role in higher grade tumour recurrence in patients with good performance status.

### REFERENCES

Bartl R, Frisch B, Fateh-Moghadam A, Kettner G, Jaeger K and Sommerfield W (1987) Histological classification and staging of multiple myeloma. A retrospective and prospective study of 674 cases. *Am J Clin Pathol* **87**: 342–355

 © Cancer Research Campaign 1997

Corwin J and Lindberg RD (1979) Solitary plasmacytoma of bone vs. extramedullary plasmacytoma and their relationship to multiple myeloma. *Cancer* **43**: 1007–1013

Fermand JP, Ravaud P, Chevert S, Leblond V, Divine M, Dreyfus F, Belanger C, Troussard X, Mariette X and Brouet JC (1995) High dose therapy and autologous blood stem cell transplantation in multiple myeloma: preliminary results of a randomized trial involving 167 patients. *Stem Cells Dayt* **13**: 156–159

Greenberg P, Parker RG, Fu YS and Abemayor E (1987) The treatment of solitary plasmacytoma of bone and extramedullary plasmacytoma. *Am J Clin Oncol* **10**: 199–204

Gromer RC and Duvall AJ (1973) Plasmacytoma of the head and neck. *J Laryngol Otol* **87**: 861–872

Guida M, Casamassima A, Abbate I, Paradiso A, Zito A, Marzullo F, Lorusso V, Timurian A, Cramarossa A and De-Lena M (1994) Solitary plasmacytoma of bone and extramedullary plasmacytoma: two different nosological entities? *Tumori* **80**: 370–377

Harwood AR, Knowling MA and Bergsagel DE (1981) Radiotherapy of extramedullary plasmacytoma of the head and neck. *Clin Radiol* **32**: 31–36

Holland J, Trenkner DA, Wasserman TH and Fineberg B (1992) Plasmacytoma – treatment results and conversion to myeloma. *Cancer* **69**: 1513–1517

Kapadia SB, Desai U and Cheng VS (1982) Extramedullary plasmacytoma of the head and neck. *Medicine* **61**: 317–329

Knowling MA, Harwood AR and Bergsagel DE (1983) Comparision of extramedullary plasmacytomas with solitary and multiple plasma cell tumors of bone. *J Clin Oncol* **1**: 255–262

Kotner LM and Wang CC (1972) Plasmacytoma of the upper air and food passages. *Cancer* **30**: 414–418

Mayr NA, Wen BC, Hussey DH, Burns CP, Staples JJ, Doornbos JF and Vigliotti AP (1990) The role of radiation therapy in the treatment of solitary plasmacytoma. *Radiother Oncol* **17**: 293–303

Mendenhall WM, Thar TL and Million RR (1980) Solitary plasmacytoma of bone and soft tissue. *Int J Radiat Oncol Biol Phys* **6**: 1497–1501

Mock PM, Neal GD and Aufdemorte TB (1987) Immunoperoxidase characterisation of extramedullary plasmacytoma of the head and neck. *Head Neck Surg* **9**: 356–361

Pahor AL (1977) Extramedullary plasmacytoma of the head and neck, paratoid and submandibular salivary glands. *J Laryngol Otol* **91**: 241–258

Petrovich Z, Fishken B, Hittle RE, Acquarelli M and Barton R (1977) Extramedullary plasmacytoma of the upper respiratory passages. *Int J Radiat Oncol Biol Phys* **2**: 723–730

Poole AG and Marchetta FC (1968) Extramedullary plasmacytoma of the head and neck. *Cancer* **22**: 14–21

Rainer EH (1970) Extramedullary plasmacytoma of upper respiratory tract. *J Laryngol Otol* **84**: 909–919

Shih LY, Dunn P, Leung WM, Chen WJ and Wang PN (1995) Localised plasmacytoma in Taiwan; comparision between extramedullary plasmacytoma and solitary plasmacytoma of bone. *Br J Cancer* **71**: 128–133

Soesan M, Paccagnella A, Chiarion-Sileni V, Salvagno L, Firnasiero A, Sotti G, Zorat AL, Faveretto A and Fiorentino M (1992) Extramedullary plasmacytoma: clinical behaviour and response to treatment. *Ann Oncol* **3**: 51–57

Todd IDH (1965) Treatment of solitary plasmacytoma. *Clin Radiol* **16**: 395–399

Wiltshaw E (1976) The natural history of extramedullary plasmacytoma and its relation to solitary myeloma of bone and myelomatosis. *Medicine* **55**: 217–238

Wiltshaw E (1978) Chemotherapy in the management of extramedullary plasmacytoma. *Cancer Chemother Pharmacol* **1**: 167–175

Woodruff RK, Whittle JM and Malpas JS (1979) Solitary plasmacytoma I: extramedullary soft tissue plasmacytoma. *Cancer* **43**: 2340–2343